# *Klebsiella pneumoniae* Carbapenemase-2 (KPC-2), Substitutions at Ambler Position Asp179, and Resistance to Ceftazidime-Avibactam: Unique Antibiotic-Resistant Phenotypes Emerge from β-Lactamase Protein Engineering

**DOI:** 10.1128/mBio.00528-17

**Published:** 2017-10-31

**Authors:** Melissa D. Barnes, Marisa L. Winkler, Magdalena A. Taracila, Malcolm G. Page, Eric Desarbre, Barry N. Kreiswirth, Ryan K. Shields, Minh-Hong Nguyen, Cornelius Clancy, Brad Spellberg, Krisztina M. Papp-Wallace, Robert A. Bonomo

**Affiliations:** a Research Service, Louis Stokes Cleveland Department of Veterans Affairs, Cleveland, Ohio, USA; b Department of Medicine, Case Western Reserve University, Cleveland, Ohio, USA; c Department of Molecular Biology and Microbiology, Case Western Reserve University, Cleveland, Ohio, USA; d Department of Pharmacology, Case Western Reserve University, Cleveland, Ohio, USA; e Department of Biochemistry, Case Western Reserve University, Cleveland, Ohio, USA; f Department of Proteomics, Case Western Reserve University, Cleveland, Ohio, USA; g Department of Bioinformatics, Case Western Reserve University, Cleveland, Ohio, USA; h Jacobs University, Bremen, Germany; i Basilea Pharmaceutica International Ltd., Basel, Switzerland; j Public Health Research Institute Center, New Jersey Medical School, Rutgers University, Newark, New Jersey, USA; k Department of Medicine, University of Pittsburgh, Pittsburgh, Pennsylvania, USA; l Los Angeles County + University California Medical Center, Los Angeles, California, USA; m Division of Infectious Diseases, Keck School of Medicine at USC, Los Angeles, California, USA; Indiana University Bloomington

**Keywords:** KPC-2, avibactam, beta-lactam, beta-lactamase, carbapenemase, ceftazidime

## Abstract

The emergence of *Klebsiella pneumoniae* carbapenemases (KPCs), β-lactamases that inactivate “last-line” antibiotics such as imipenem, represents a major challenge to contemporary antibiotic therapies. The combination of ceftazidime (CAZ) and avibactam (AVI), a potent β-lactamase inhibitor, represents an attempt to overcome this formidable threat and to restore the efficacy of the antibiotic against Gram-negative bacteria bearing KPCs. CAZ-AVI-resistant clinical strains expressing KPC variants with substitutions in the Ω-loop are emerging. We engineered 19 KPC-2 variants bearing targeted mutations at amino acid residue Ambler position 179 in *Escherichia coli* and identified a unique antibiotic resistance phenotype. We focus particularly on the CAZ-AVI resistance of the clinically relevant Asp179Asn variant. Although this variant demonstrated less hydrolytic activity, we demonstrated that there was a prolonged period during which an acyl-enzyme intermediate was present. Using mass spectrometry and transient kinetic analysis, we demonstrated that Asp179Asn “traps” β-lactams, preferentially binding β-lactams longer than AVI owing to a decreased rate of deacylation. Molecular dynamics simulations predict that (i) the Asp179Asn variant confers more flexibility to the Ω-loop and expands the active site significantly; (ii) the catalytic nucleophile, S70, is shifted more than 1.5 Å and rotated more than 90°, altering the hydrogen bond networks; and (iii) E166 is displaced by 2 Å when complexed with ceftazidime. These analyses explain the increased hydrolytic profile of KPC-2 and suggest that the Asp179Asn substitution results in an alternative complex mechanism leading to CAZ-AVI resistance. The future design of novel β-lactams and β-lactamase inhibitors must consider the mechanistic basis of resistance of this and other threatening carbapenemases.

## IMPORTANCE

Antibiotic resistance is emerging at unprecedented rates and threatens to reach crisis levels. One key mechanism of resistance is the breakdown of β-lactam antibiotics by β-lactamase enzymes. KPC-2 is a β-lactamase that inactivates carbapenems and β-lactamase inhibitors (e.g., clavulanate) and is prevalent around the world, including in the United States. Resistance to the new antibiotic ceftazidime-avibactam, which was designed to overcome KPC resistance, had already emerged within a year. Using protein engineering, we uncovered a mechanism by which resistance to this new drug emerges, which could arm scientists with the ability to forestall such resistance to future drugs.

## INTRODUCTION

Antibiotic resistance is becoming an international health care crisis (1). Among the drug-resistant phenotypes expressed by Gram-negative bacteria, resistance to β-lactam antibiotics mediated by β-lactamases is the most problematic. The carbapenemases, β-lactamases that inactivate carbapenems (imipenem, meropenem, ertapenem, and doripenem), are considered by the Centers for Disease Control and Prevention to be among the major threats facing health care providers (2). Of the prevalent carbapenemases (KPC-2, KPC-3, NDM, OXA-23, OXA-24/40, and OXA-48), the KPCs are among the most widespread (3). Since the discovery and structural elucidation of KPC-2, much attention has been focused on mechanistic explanations of why this class A carbapenemase is resistant to inactivation by the β-lactamase inhibitors clavulanate, sulbactam, and tazobactam (4–6). The welcome introduction of avibactam, a diazabicyclooctane (DBO) non-β-lactam β-lactamase inhibitor which can inactivate KPC-2, was a major advance in therapy. As a result of the formulation of ceftazidime-avibactam (CZA), many clinical isolates bearing KPC-2 or KPC-3 were rendered susceptible, offering a potential alternative to undesirable antibiotics, such as polymyxins and tigecycline, which are more toxic and/or less effective than other antibiotics.

The KPC carbapenemase demonstrates a broad substrate profile, including penicillins, cephalosporins, carbapenems, and β-lactamase inhibitors (5). The effects of several amino acid substitutions were previously studied in KPC-2 and revealed that most substitutions increase susceptibility to β-lactamase inhibitors and β-lactams, indicating that this carbapenemase is highly optimized to hydrolyze a wide variety of compounds (7–11).

**FIG 1:**
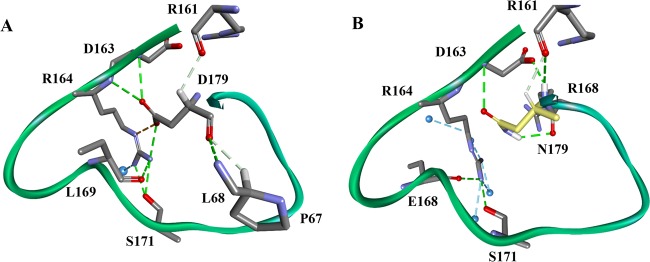
Ω-Loop hydrogen bond networking changes due to the aspartate (D)-to-asparagine (N) substitution at Ambler position 179 in KPC-2. (A) KPC-2. (B) Asp179Asn (D179N) variant.

One exception has been the study of substitutions at position Arg164 in the Ω-loop (7). Normally, the arginine at this position forms a conserved salt bridge with an aspartic acid at position 179 in class A β-lactamases (7) (Fig. 1A). To understand the structural significance of this salt bridge, site-saturation mutagenesis was undertaken. Surprisingly, we discovered that amino acid substitutions at Ambler position 164 increased the MICs for ceftazidime and the ceftazidime-avibactam combination even before clinical variants demonstrated ceftazidime-avibactam resistance (7, 12). These observations suggested that the Arg164Ser variant of KPC-2 has an increased capacity to hydrolyze ceftazidime. Further studies demonstrated that substitutions of alanine, glutamine, and asparagine at position 179 break this salt bridge and confer increased resistance to ceftazidime and resistance to the ceftazidime-avibactam combination (12).

What is the biochemical basis of this phenotype? Structural analysis suggested that Arg164 forms hydrogen bond interactions with other residues within the Ω-loop (Fig. 1A). In contrast, the C-terminal amino acid of the Ω-loop (Asp179) displays a more extensive pattern of hydrogen bonds in the KPC β-lactamase, including hydrogen bonds to residues outside the Ω-loop (Pro67, Leu68, and Arg161) (Fig. 1A). Therefore, the effects of amino acid substitutions at position 179 may have implications beyond the Ω-loop dynamics and may be different from those seen at position 164. Building upon knowledge gained from studies of Arg164 and the resistance to ceftazidime-avibactam that is being presently reported in the clinic (2, 13, 14), we were compelled to attempt to understand how amino acid substitutions at Asp179 of KPC-2 impact the enzymatic mechanism, with an emphasis on ceftazidime-avibactam resistance. Our investigations have particular significance, as ceftazidime-avibactam-nonsusceptible Asp179Tyr variants in KPC-3 pose a major clinical challenge (2, 15).

## RESULTS AND DISCUSSION

### Substitutions at position Asp179 alter KPC-2 β-lactamase expression in *Escherichia coli*.

**FIG 2:**
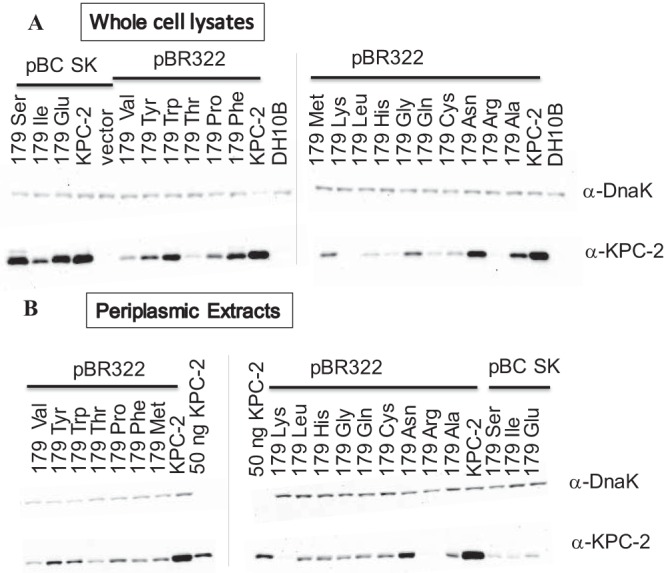
Western blot of whole-cell preparations (A) and periplasmic extracts (B) of KPC-2 Asp179 variants in *E. coli* DH10B. vector, DH10B cells containing pBC SK vector; DH10B, unaltered cells. All variants are in the pBR322 vector except pBC SK-Ser, pBC SK-Ilu, and pBC SK-Glu.

All 19 amino acid variants at Ambler position 179 in KPC-2 β-lactamase were engineered. To determine whether these single substitutions alter protein expression, immunoblots using whole-cell preparations and periplasmic extracts were probed with an anti-KPC-2 polyclonal antibody (Ab) that is sensitive and specific and maps to three main linear epitopes of KPC-2 (9). Single amino acid substitutions at position Asp179 in the Ω-loop generally result in decreased levels of expression (Fig. 2), possibly due to differences in overall protein stability. However, certain variants (Asp179Asn) maintain notable levels of expression during the exponential phase of the growth curve.

### Microbiological analysis. (i) Asp179 variants of the Ω-loop of KPC-2 and β-lactam resistance.

The impact of site-saturation mutagenesis at Ambler position 179 of KPC-2 on antibiotic resistance was next assessed using whole-cell viability assays. Twenty-four different β-lactam and β-lactam–β-lactamase inhibitor combinations were tested for susceptibility against KPC-2 and the 19 variants expressed in *E. coli* DH10B cells (Tables 1 and 2). The *Klebsiella pneumoniae* KPC-2-containing positive-control strain maintained resistance (as defined by Clinical and Laboratory Standards Institute [CLSI] criteria) against all the commercially available β-lactams tested (Table 1). The *E. coli* strain containing the KPC-2 construct exhibited resistance to the same panel of tested antibiotics, including cephalosporins, monobactams, and carbapenems (Table 1). In contrast, the Asp179 variants (except Asp179Asn) expressed in *E. coli* generally showed an increase in susceptibility to the β-lactam antibiotics, possibly attributable to the attenuated protein expression of the variants (Fig. 2). Notably, striking resistance to ceftazidime was maintained by all the variants, including the least-expressed Asp179Lys and Asp179Arg variants (MICs of 64 µg/ml for both variants) (Table 1).

**TABLE 1:** MICs of various β-lactam and β-lactam–β-lactamase inhibitors and combinations against KPC-2 Asp179 variants^a^

Strain	MIC(s) (µg/ml)																	
	CAZ	THIN	TAX	FEP	CRO	TAR	TOL-TAZ^b^	AMP	PIP	AMP-SUL^c^	AMP-CLA^c^	PIP-TAZ^d^	AZT	BAL	IMI	ERT	DOR	MEM
*K. pneumoniae* KPC-2^e^	64	2,048	64	64	>512	512	32	>8,192	2,048	>256	64	512/64	256	8	16	32	8	16
																		
*E. coli* DH10B	0.25	4	≤0.06	≤0.125	≤0.06	0.06	0.25	8	2	≤0.06	≤0.03	2/0.25	0.06	≤0.06	0.5	≤0.03	≤0.06	≤0.06
*E. coli* DH10B pBC SK	0.25	4	≤0.06	≤0.125	≤0.06	<0.06	0.25	4	2	≤0.06	≤0.03	2/0.25	0.06	≤0.06	0.5	≤0.03	≤0.06	≤0.06
*bla*_SHV1_	8	256	0.25	2	0.25	64	2	>8,192	4,096	>256	8	512/64	0.5	0.25	1	32	0.125	0.06
*bla*_KPC-3_	32	128	4	4	8	32	8	1,024	128	128	16	128/16	32	0.25	2	0.25	0.125	0.125
*bla*_KPC-2__179__Asp (WT)_	**1**	**64**	**0.25**	**0.125**	**0.5**	**1**	**1**	**64**	**8**	**0.125**	**0.125**	**8/1**	**2**	**≤0.06**	**1**	**≤0.06**	**0.125**	**0.06**
*bla*_179__Glu_	512	32	0.5	1	1	8	8	256	8	8	1	8/1	16	0.25	1	0.06	0.125	0.06
*bla*_179__Ile_	128	8	1	2	2	16	8	8	32	≤0.06	≤0.03	8/1	1	0.06	0.5	0.06	0.06	≤0.06
*bla*_179__Ser_	32	32	2	2	2	16	8	128	32	≤0.06	0.25	8/1	0.5	0.125	0.5	0.125	0.125	0.06
																		
*E. coli* DH10B pBR322 *bla*_KPC-2 179 Asp (WT) _	**128**	**1,024**	**32**	**64**	**128**	**256**	**32**	**8,192**	**2,048**	**>256**	**64**	**1,024/128**	**256**	**2**	**8**	**8**	**4**	**4**
*bla*_179__Ala_	512	32	4	8	8	32	16	64	128	≤0.06	≤0.03	16/2	4	0.5	1	0.06	0.125	0.06
*bla*_179__Arg_	64	16	0.5	1	1	2	8	128	8	0.25	0.06	8/1	0.5	0.25	1	0.06	0.125	0.06
(Asp179Asn) *bla*_179__Asn_	512	512	32	16	64	128	64	8,192	1,024	>256	64	512/64	256	1	4	2	4	2
*bla*_179__Cys_	128	128	4	4	8	32	16	1,024	64	16	8	16/2	16	0.25	1	0.25	0.125	0.125
*bla*_179__Gln_	128	64	2	4	8	8	8	256	32	8	0.5	16/2	2	0.25	1	0.125	0.125	0.06
*bla*_179__Gly_	512	64	16	16	32	32	16	64	256	≤0.06	≤0.06	16/2	16	1	1	0.25	0.125	0.06
*bla*_179__his_	512	16	4	4	8	16	16	64	64	≤0.06	≤0.03	16/2	4	0.25	1	0.125	0.125	0.06
*bla*_179__Leu_	512	32	8	8	16	32	16	32	128	≤0.06	≤0.03	16/2	4	0.5	1	0.06	0.125	0.125
*bla*_179__Lys_	64	8	0.5	0.5	1	2	16	32	8	≤0.06	≤0.03	8/1	0.5	0.25	1	0.06	0.125	0.06
*bla*_179__met_	>512	32	8	16	32	32	16	32	128	≤0.06	≤0.03	16/2	8	0.5	1	0.25	0.125	0.06
*bla*_179__Phe_	512	16	16	16	32	16	16	16	128	≤0.06	≤0.03	16/2	8	0.5	1	0.125	0.125	0.06
*bla*_179__pro_	512	16	2	4	4	16	16	16	32	≤0.06	≤0.03	16/2	2	0.25	1	0.125	0.125	0.06
*bla*_179__Thr_	512	32	4	4	8	16	16	64	64	≤0.06	≤0.03	16/2	2	0.25	1	0.125	0.125	0.06
*bla*_179__Trp_	>512	16	16	16	32	32	16	16	128	≤0.06	≤0.03	16/2	8	0.5	0.5	0.125	0.125	0.06
*bla*_179__Tyr_	>512	32	16	16	32	32	16	16	128	≤0.06	≤0.03	16/2	8	0.5	0.5	0.25	0.125	0.06
*bla*_179__Val_	512	16	8	8	16	32	16	32	128	≤0.06	≤0.03	16/2	4	0.5	1	0.25	0.125	0.06

a Data for *E. coli* strains containing wild-type KPC-2 in pBC SK and pBR322 vectors are boldface. AMP, ampicillin; AZT, aztreonam; BAL, BAL30072; CAZ, ceftazidime; CLA, clavulanic acid; CRO, ceftriaxone; DOR, doripenem; ERT, ertapenem; FEP, cefepime; IMI, imipenem; MEM, meropenem; PIP, piperacillin; SUL, sulbactam; TAR, ceftaroline; TAX, cefotaxime; TAZ, tazobactam; THIN, cephalothin; TOL, ceftolozane; WT, wild type.

b Ceftolozane-tazobactam was tested at a ratio of 2:1.

c Ampicillin was held at a constant 50 µg/ml.

d Pipercillin-tazobactam was tested at a ratio of 8:1.

e Control strain producing TEM-1 and SHV-29.

### (ii) Addition of avibactam overcomes the ceftazidime resistance mediated by KPC-2 but not that mediated by the Asp179 variants.

The addition of the β-lactamase inhibitor avibactam abrogated ceftazidime resistance in the KPC-2-containing strain but, alarmingly, was insufficient to restore susceptibility to any of the strains harboring the Asp179 variants (Table 2). This supported an earlier observation that the Asp179Ala, Asp179Gln, and Asp179Asn variants of KPC-2 expressed in *E. coli* conferred resistance to ceftazidime-avibactam (12).

**TABLE 2:**
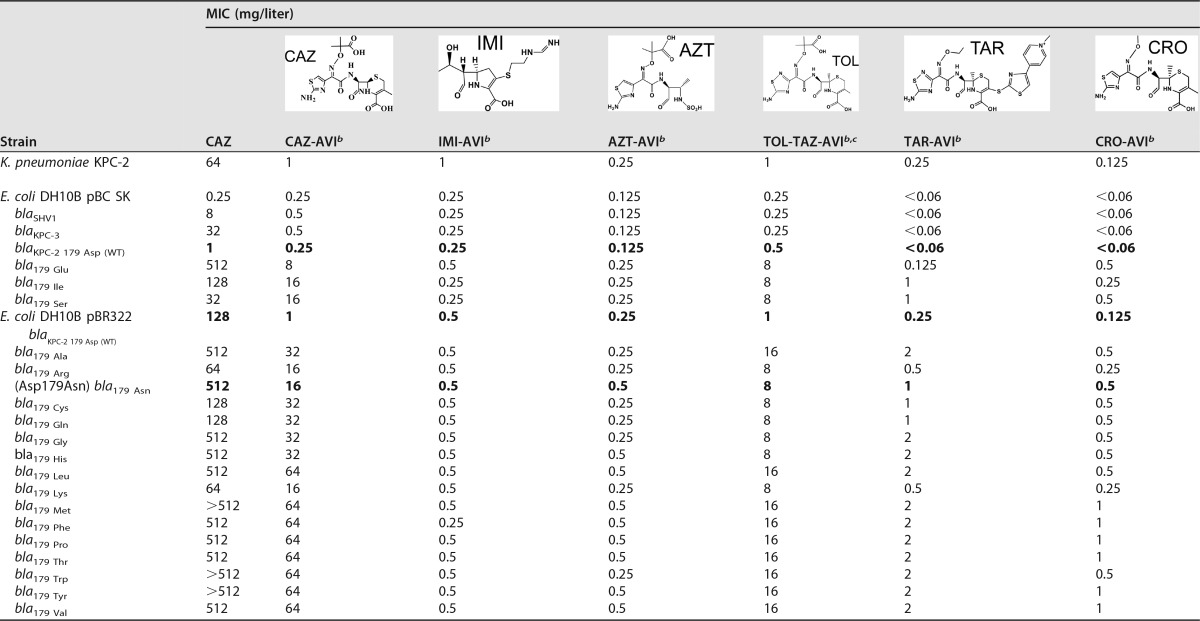
MICs of various β-lactam and non-β-lactam–β-lactamase inhibitors and combinations against *E. coli* strains containing KPC-2 Asp179 variants^a^

a Data for *E. coli* strains containing wild-type KPC-2 in pBC SK and pBR322 vectors are boldface. AVI, avibactam; AZT, aztreonam; CRO, ceftriaxone; IMI, imipenem; TAR, ceftaroline; TAZ, tazobactam; TOL, ceftolozane.

b Avibactam was held constant at 4 mg/liter.

c Ceftolozane-tazobactam was tested at a ratio of 2:1.

### (iii) The Asp179Asn variant exhibits a resistant antimicrobial profile.

The strain harboring the Asp179Asn variant stood out among the other 18 variant strains for conferring levels of resistance to all commercially available β-lactams tested as monotherapies except meropenem (the breakpoint for BAL30072 is not yet defined), similarly to the resistance profile of KPC-2 (Table 1). Notably, the Asp179Asn variant strain demonstrated elevated resistance to ceftazidime (KPC-2 was measured at 128 µg/ml compared to Asp179Asn measured at 512 µg/ml) (Table 1) and to the ceftazidime-avibactam combination (KPC-2 measured at 1 µg/ml versus Asp179Asn measured at 16 µg/ml) (Table 2).

### Aztreonam-avibactam and ceftaroline-avibactam, two combinations currently in clinical trials, effectively showed lower MICs for the Asp179Asn strain.

To gain insight into the therapeutic potential of clinically relevant avibactam combinations, susceptibility testing against commercially available avibactam (Advanced ChemBlocks) combined with aztreonam and ceftaroline was conducted on strains containing KPC-2 and each of the variants. Both the KPC-2 and Asp179Asn-containing strains were resistant to each of these β-lactams in the absence of avibactam (Table 1). Avibactam restored the susceptibility of KPC-2 to aztreonam and ceftaroline based on the breakpoint for the each β-lactam alone. In contrast to the resistance to ceftazidime-avibactam of the Asp179Asn strain, ceftaroline-avibactam decreased the resistance of the Asp179Asn strain to an intermediate level based on the breakpoint of ceftaroline alone (Table 2). The aztreonam-avibactam combination was even more effective, rendering the Asp179Asn strain susceptible to aztreonam. These data suggest that ceftaroline and aztreonam may be attractive and viable therapeutic partners for avibactam against Asp179Asn variants of KPC-2.

### Avibactam in combination with imipenem and ceftriaxone lowers the drug MICs of the Asp179Asn strain; the structure of the R1 side chain in ceftazidime contributes to ceftazidime-avibactam resistance.

**FIG 3:**
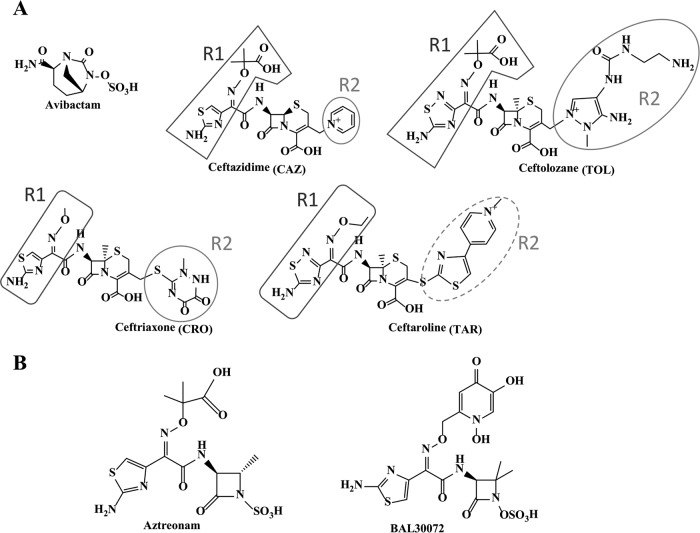
Structures of β-lactams and β-lactamase inhibitors. The R1 groups are encompassed by boxes, and circles are used to surround the R2 groups. The dotted line indicates an R2 group that is observed intact when bound to D179N (Fig. S2).

To further explore the structure-activity factors (particularly Asp179Asn) responsible for the increased ceftazidime resistance of the Asp179 variant β-lactamases, experiments using avibactam in combination with a representative carbapenem (imipenem), a cephalosporin structurally similar to ceftazidime (ceftolozane; tested in combination with tazobactam), and a cephalosporin structurally distinct from ceftazidime (ceftriaxone) were conducted with strains containing KPC-2 and each of the variants. Ceftolozane, a novel cephalosporin, is the β-lactam most similar in structure to ceftazidime (Fig. 3). Ceftazidime and ceftolozane differ by only one atom in the R1 side chain, with ceftazidime possessing a carbon atom (aminothiazole) and ceftolozane a nitrogen atom (aminothiadiazole). Ceftriaxone is an expanded-spectrum oxyimino-cephalosporin that has been in commercial use for decades. Ceftazidime and ceftriaxone share the same aminothiazole group but differ on the oxyimino end of R1 (Fig. 3). Ceftriaxone has a less bulky oxyimino group and lacks the acidic carboxylate group (like the oxyimino group of ceftaroline).

Both the KPC-2 and Asp179Asn-containing strains were resistant to imipenem, ceftolozane-tazobactam, and ceftriaxone in the absence of avibactam (Table 1). With results mimicking the susceptibility to ceftazidime-avibactam, avibactam restored susceptibility of KPC-2 to imipenem, ceftolozane-tazobactam, and ceftriaxone based on the breakpoints for the respective β-lactams. The Asp179Asn strain maintained resistance to ceftolozane-tazobactam-avibactam (based on the breakpoint of ceftolozane-tazobactam), similarly to the ceftazidime-avibactam resistance results (Table 2). In contrast, avibactam restored the susceptibility of the Asp179Asn strain to ceftriaxone and imipenem. Ceftriaxone-avibactam was the most effective cephalosporin combination tested (Table 2), suggesting that the bulkiness and/or the carboxylate group on R1 is the structural moiety that may be primarily responsible for ceftazidime resistance.

### Biochemical analysis. (i) The Asp179Asn variant hydrolyzes ceftazidime slowly but demonstrates a lower *K*_*i*_ for ceftazidime.

**FIG 4:**
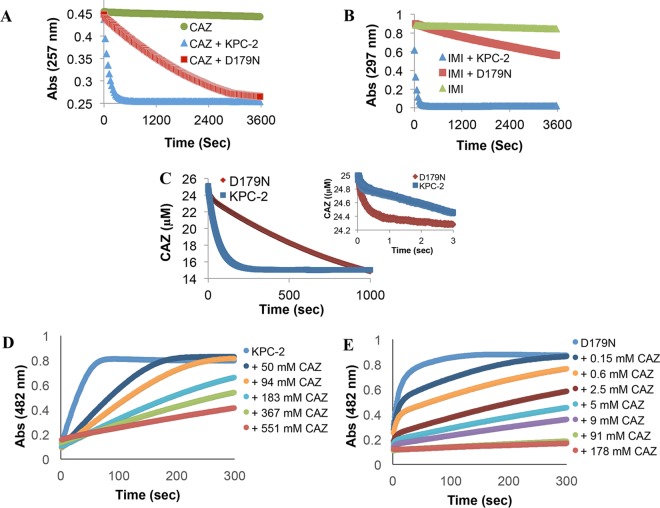
(A) KPC-2 and Asp179Asn (D179N) (1 µM enzyme) hydrolysis of 25 µM ceftazidime (CAZ) at room temperature. (B) KPC-2 and Asp179Asn (0.5 µM enzyme) hydrolysis of 100 µM imipenem (IMI) at room temperature. (C) Examining pre-steady-state kinetics using a stopped-flow apparatus and hydrolysis of 25 µM ceftazidime by 2 µM KPC-2 or and Asp179Asn variant at 25°C for 1,000 s and (inset) 3 s. (D and E) Competitive inhibition curves determined with 50 µM nitrocefin and increasing concentrations of CAZ with 7 nM KPC-2 (apparent *K*_*i*_, 3.5 mM) (D) and 425 nM Asp179Asn (apparent *K*_*i*_, 0.13 mM) (E) at room temperature.

To elucidate the mechanistic differences between Asp179Asn and KPC-2, each was purified for biochemical analysis. In previous comparisons of KPC-2 to Asp179Asn performed using periplasmic extracts, we noted similar rates of ceftazidime hydrolysis (12). However, the previous analysis was performed using amounts of β-lactamase normalized for nitrocefin (NCF) hydrolysis. Here, we used 1 μM KPC-2 and Asp179Asn to measure the hydrolysis of ceftazidime and used a 0.5 μM concentration of each enzyme in the assessment of imipenem hydrolysis. The Asp179Asn variant hydrolyzed both β-lactams at a much lower rate than KPC-2 (Fig. 4A and B).

We previously reported a burst in hydrolysis of ceftazidime by KPC-2 (7). Thus, the conditions were optimized for assessment of early time points in the hydrolysis of ceftazidime by KPC-2 compared to Asp179Asn. A burst amplitude of 0.16 µM ± 0.02 µM was obtained with 2 µM KPC-2 and 25 µM ceftazidime (similarly to our previous results with KPC-2) (Fig. 4C). A higher burst amplitude of 0.57 µM ± 0.06 µM was obtained with Asp179Asn under the same conditions. A burst that occurs prior to the establishment of the linear steady-state reaction reflects rapid acylation followed by a relatively slow rearrangement or product formation (equation 1) (16) as follows:

(1) $$E+S\;\overset{k_{ac}}{\to }\;E\text{-}I\;\overset{k_{2}}{\to }\;E\cdot P\to E+P$$

In such a reaction, the burst decay constant, *k*_burst_, is given by *k*_burst__=_ *k*_*ac*_ + *k*_2_; the burst amplitude, *A*, is given by *A* = [Eo] ⋅ (*k*_*ac*_/*k*_*ac*_ + *k*_2_)^2^, where [Eo] is the concentration of active enzyme; and the steady-state rate, *k*_ss_, is given by *k*_ss_ = *k*_2_/(1 + *k*_2_/*k*_*ac*_).

For the experiment represented in Fig. 3, the values were calculated to be *k*_*ac*_ = 0.043 ± 0.004 s^−1^ and *k*_2_ = 0.097 ± 0.010 s^−1^ for KPC-2 and *k*_*ac*_ = 0.038 ± 0.004 s^−1^ and *k*_2_ = 0.030 ± 0.003 s^−1^ for the Asp179Asn variant. Thus, multiple steps along the reaction coordinate are likely affected by the substitution, although the effect on the hydrolysis of the acyl intermediate or product release (*k*_2_) appears to be the more profound.

To compare the apparent affinity of ceftazidime for KPC-2 to its affinity for the Asp179Asn variant, we used various concentrations of ceftazidime to inhibit hydrolysis of a reporter substrate, nitrocefin (Fig. 4D and E). More ceftazidime was required with the wild type (apparent *K*_*i*_, 3.5 mM) to reach the same level of inhibition as that seen with the Asp179Asn variant (apparent *K*_*i*_, 0.13 mM), as is expected in a reaction such as that described in equation 1 in which the *k*_2_ value is lower for the variant.

### (ii) The Asp179Asn variant is a “trap” for β-lactams.

To further support our kinetic analysis, timed mass spectrometry was used to probe for mechanistic differences between KPC-2 and the Asp179Asn variant. The β-lactamases (E) were incubated with a substrate (S [ceftazidime, imipenem, or aztreonam]) and an inhibitor (I [avibactam]) at a molar ratio of 1:1:1 (E:S:I), thus establishing a direct competition between the β-lactam (avibactam) and the β-lactamase.

**FIG 5:**
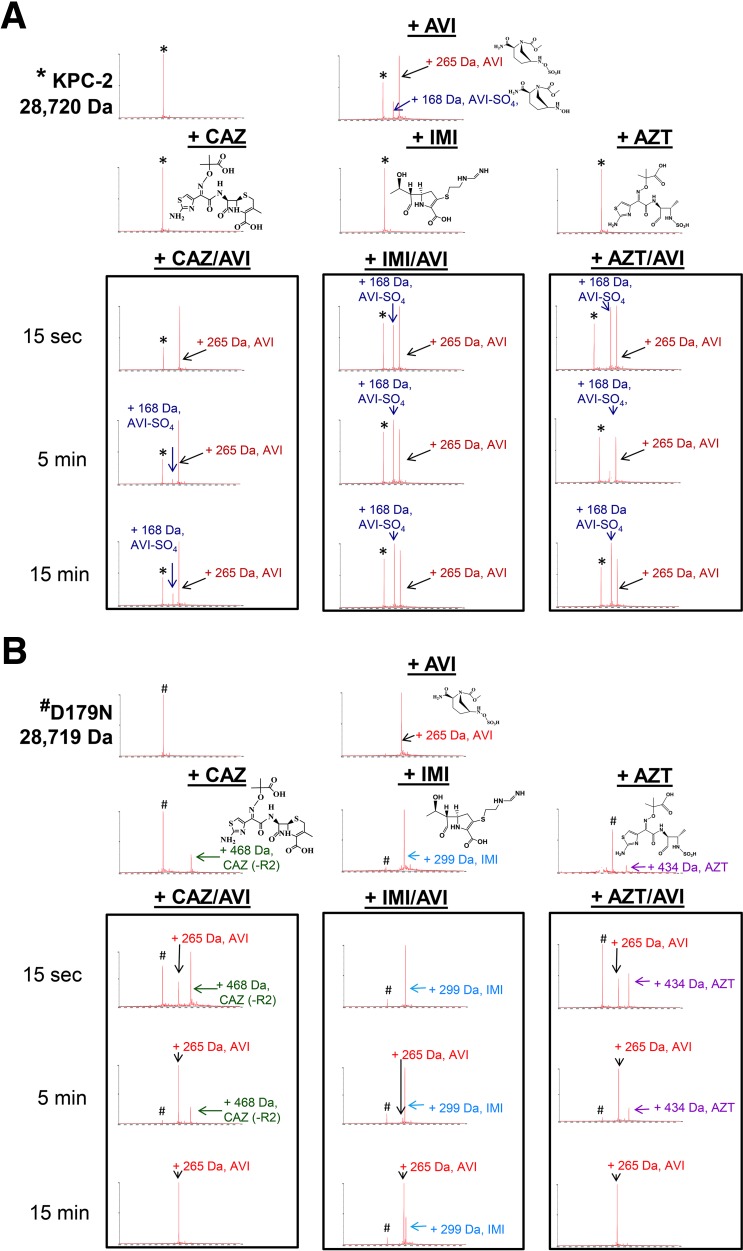
(A) Mass spectrometry of KPC-2 (8.7 µM) reacted with avibactam (AVI) ± ceftazidime (CAZ), imipenem (IMI), or aztreonam (AZT). The enzyme/substrate/inhibitor molar ratio was held constant at 1:1:1 at room temperature. Controls were incubated for 5 min. *, KPC-2 alone, mass of 28,720 Da. (B) Mass spectrometry of Asp179Asn (D179N) (8.7 µM) incubated with avibactam (AVI) ± ceftazidime (CAZ), imipenem (IMI), or aztreonam (AZT). The enzyme/substrate/inhibitor molar ratio was held constant at 1:1:1 at room temperature. Controls were incubated for 5 min. #, D179N alone, mass of 28,719 Da.

Several characteristics of the Asp179Asn variant that are distinct from those of the wild-type enzyme were revealed. KPC-2 preferentially bound avibactam compared to the tested β-lactams (Fig. 5A; see also Fig. S1 in the supplemental material). This observation was not surprising as avibactam is a potent inhibitor of KPC-2. Also, ceftazidime, imipenem, and aztreonam are substrates for KPC-2 and could therefore be hydrolyzed before they could be detected as mass adducts. In contrast, under the same conditions, Asp179Asn preferentially bound all the tested β-lactams compared to avibactam (Fig. 5B and S1).

10.1128/mBio.00528-17.1 FIG S1  Mass spectrometry of KPC-2 and Asp179Asn (D179N) incubated with avibactam ± ceftazidime, imipenem, or aztreonam. The enzyme/substrate/inhibitor molar ratio was held constant at 1:1:1 at room temperature. Controls were incubated for 5 min. The peak percentages show generalized trends in complex formations. Download FIG S1, TIF file, 0.2 MB. Copyright © 2017 Barnes et al. 2017 Barnes et al. This content is distributed under the terms of the Creative Commons Attribution 4.0 International license.

Asp179Asn binds imipenem for the longest time, with the acyl-enzyme remaining the predominant species of Asp179Asn at 15 min, unlike ceftazidime or aztreonam (the acyl-enzymes are undetectable by 15 min). These data suggest that the asparagine substitution at the 179 aspartate position allows the Asp179Asn β-lactamase to “trap” the substrate. The decrease in the rate of deacylation, predicted by the transient kinetic analysis described above, may be sufficient to explain the preferential trapping of β-lactams compared to avibactam. This step is not involved in the interaction of avibactam with the β-lactamase (17); therefore, its reaction kinetics are not so strongly affected.

### (iii) Desulfation of avibactam is unique to KPC-2 and is not observed with the Asp179Asn variant.

Desulfation of avibactam is known to occur with KPC-2 (18, 19), and we show here that it does not occur in the Asp179Asn variant (Fig. 5A and S1). Possible explanations for the distinct ability of KPC-2 to desulfate avibactam among the β-lactamases include a lack of hydrogen bonds with the N6 atom and a necessary water molecule that desulfates avibactam (18). Both the avibactam and desulfated avibactam bound to KPC-2 are stable complexes still present at 48 h, although the apo-KPC-2 form becomes more predominant (data not shown).

### (iv) Mass spectrometry and the detection of a unique mass adduct.

Interestingly, the reaction of Asp179Asn with ceftazidime produced two protein charge envelopes; one was the expected primary charge envelope at approximately 1,100 to 2,000 *m*/*z* (29,187 Da mass, corresponding to the predicted mass of 28,719 Da for Asp179Asn in addition to the 468 Da for ceftazidime minus R2), and the second was a more highly charged species (at approximately 800 to 1,200 *m*/*z*) with a different overall deconvoluted mass that was not observed with KPC-2 (Fig. S4; Table 3). The formation and elimination of this secondary envelope were time dependent, deconvoluted to a single protein peak with a mass of 29,124 Da, and were unique to Asp179Asn reacted with ceftazidime or the ceftazidime-avibactam combination. The identity of this +405 adduct with a particular chemical rearrangement in ceftazidime is not obvious. We did not observe this peak with avibactam in combination with aztreonam or imipenem (data not shown), eliminating the likelihood of a mass spectrometry artifact. We take this observation to suggest that the Asp179Asn-ceftazidime complex may undergo an alternative or additional conformational change during hydrolysis, resulting in an altered surface charge and a corresponding shift in the mass-to-charge ratio.

**TABLE 3:** Observed masses (predicted and unexpected) of Asp179Asn (D179N) and KPC-2 β-lactamases as apo-enzymes and adducted to the non-β-lactam–β-lactamase inhibitor avibactam and to various β-lactams^a^

Mass category and β-lactamase or adduct	Change in mass (Da)	Mass (Da)	
		D179N	KPC-2
Predicted			
Enzyme	0	28,719	28,720
+AVI	+265	28,984	28,985
+AVI (−SO_4_)	+168	ND	28,888
+CAZ (−R2)	+468	29,187	ND
+CRO (−R2)	+395	29,114	ND
+TOL (−R2)	+468	29,187	ND
+TAR	+606	29,325	ND
+BAL30072	+518	29,237	ND
+IMI	+299	29,018	ND
+AZT	+434	29,153	ND
Unique (for adducts in a second charge envelope)			
+CAZ (−R2) −63 Da	+405	29,124	ND
+CRO (−R2) −61 Da	+334	29,053	ND

a ND, not detected.

In an attempt to understand the mechanistic basis for the formation of the +405 adduct, structurally similar cephalosporins (ceftriaxone, ceftolozane with tazobactam, and ceftaroline) were tested with the Asp179Asn variant. The mass adducts for Asp179Asn and ceftazidime, ceftriaxone and ceftolozane, which have good leaving groups in R2, were consistent with the molecular weights of the antibiotics after elimination of the R2 group (Fig. S2; Table 3). Ceftaroline, which does not have a good R2 leaving group, bound to Asp179Asn with an intact R2 group (Fig. S2). Ceftriaxone and Asp179Asn revealed the expected primary envelope with a deconvoluted mass of 29,114 Da (predicted mass of Asp179Asn of 28,719 Da in addition to the 395 Da of ceftriaxone minus R2) and a second charge envelope which corresponded to a unique mass adduct of 29,053 Da (Fig. S5; Table 3). This adduct equates to ceftriaxone minus 61 Da, which parallels the mass of ceftazidime missing 63 Da in the Asp179Asn-ceftazidime adduct. These data suggest that a time-dependent modification to the antibiotic occurs and is selective for Asp179Asn with ceftazidime and ceftriaxone. The nature and clinical impact of this modification are being explored.

10.1128/mBio.00528-17.2 FIG S2  Mass spectrometry of KPC-2 or Asp179Asn (D179N) (8.7 µM) incubated with ceftriaxone (CRO), ceftolozane (TOL)-tazobactam, and ceftaroline (TAR). The enzyme/substrate molar ratio was held constant at 1:1 at room temperature. These experiments were performed in the absence of avibactam. *, KPC-2 alone, mass of 28,720 Da; #, D179N alone, mass of 28,719 Da. Download FIG S2, TIF file, 0.1 MB. Copyright © 2017 Barnes et al. 2017 Barnes et al. This content is distributed under the terms of the Creative Commons Attribution 4.0 International license.

10.1128/mBio.00528-17.3 FIG S3  Mass spectrometry of KPC-2 and Asp179Asn (D179N) (8.7 µM) incubated with BAL30072 at a 1:1 molar ratio at room temperature. *, KPC-2 alone, mass of 28,720 Da; #, D179N alone, mass of 28,719 Da. Download FIG S3, TIF file, 0.1 MB. Copyright © 2017 Barnes et al. 2017 Barnes et al. This content is distributed under the terms of the Creative Commons Attribution 4.0 International license.

10.1128/mBio.00528-17.4 FIG S4  Mass spectrometry of KPC-2 and Asp179Asn (D179N) (8.7 µM) incubated with ceftazidime, avibactam, or the combination at a 1:1:1 molar ratio at room temperature. Bold arrows point to a second charge envelope, which deconvolutes to a single protein peak with a mass of 29,124 Da. Spectrum of both charge envelopes deconvoluted together is shown on the right. Presence of the 29,124 Da peak is graphed above. Download FIG S4, TIF file, 0.1 MB. Copyright © 2017 Barnes et al. 2017 Barnes et al. This content is distributed under the terms of the Creative Commons Attribution 4.0 International license.

10.1128/mBio.00528-17.5 FIG S5  Mass spectrometry of KPC-2 and Asp179Asn (D179N) (8.7 µM) incubated with ceftriaxone (CRO), avibactam, or the combination at a 1:1:1 molar ratio at room temperature. Arrows point to a second charge envelope, which deconvolutes to a single protein peak with a mass of 29,053 Da. Download FIG S5, TIF file, 0.1 MB. Copyright © 2017 Barnes et al. 2017 Barnes et al. This content is distributed under the terms of the Creative Commons Attribution 4.0 International license.

### (v) BAL30072, a monosulfactam, lowers the drug MICs of the ceftazidime-avibactam (CZA)-resistant Asp179 variants.

Given the levels of resistance attributed to the single-amino-acid substitutions at position Asp179 in KPC-2, we were compelled to test the novel monosulfactam BAL30072 for its efficacy against these variants (20). BAL30072, like aztreonam, consists of a monocyclic β-lactam scaffold with an R1 group containing a siderophore moiety and a thiazole ring similar to that in the R1 group of ceftazidime (Fig. 3). Aztreonam was previously shown to have a 927-fold-higher *k*_cat_ value and a 3,031-fold-higher *k*_cat_*/K*_*m*_ value for KPC-2 than BAL30072 (20). Bypassing KPC-2 through a lack of positive interactions (high *K*_*m*_) and targeting penicillin-binding proteins (PBP) is therefore likely responsible for the potent activity of BAL30072 against strains expressing KPC-2.

Here, we found potent activity of BAL30072 against the Asp179 variants of KPC-2 (Table 1). In addition, we conducted mass spectrometry with KPC-2 and the Asp179Asn variant with BAL30072 (Fig. S3). The Asp179Asn variant bound BAL30072 for longer than an hour (longer than a typical bacterial division cycle) and longer than any other antibiotic tested, while KPC-2 did not bind BAL30072 at all. MIC analyses of BAL30072 against KPC-2 and Asp179Asn resulted in the lowest MICs of all the antibiotics tested (see Table 1). In addition, BAL30072 inhibits penicillin-binding proteins (PBP 1a, PBP 1b, and PBP 3) (20). On the basis of these observations, BAL30072 could be effective with strains containing Asp179Asn and is also “trapped” in the active site of Asp179Asn. The Asp179Asn trapping of this monosulfactam (aztreonam), cephalosporins, and carbapenems suggests that trapping is not reserved for select classes or activities of β-lactams.

### (vi) Molecular modeling of KPC-2 and Asp179Asn with and without ceftazidime; the Asp179Asn Ω-loop is more flexible and mobile.

**FIG 6:**
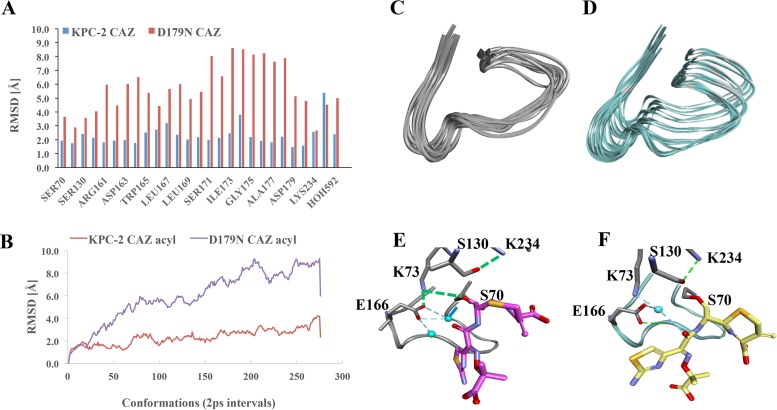
Conformational assessment of the molecular docking of ceftazidime in the acyl complex. (A) Root mean square deviations (RMSD) of Ω-loop residue data in Asp179Asn (D179N) and KPC-2. (B) Subangstrom movement (atomic RMS fluctuations) of each atom due to thermal energy during MDS. (C) Ω-Loop conformations of KPC-2 (C) and Asp179Asn (D). Molecular docking of KPC-2 (E) and Asp179Asn (F) with ceftazidime as acyl-enzyme during a 550-ps MDS analysis. Ω-Loops and deacylation waters are shown, but ceftazidime has been omitted from the image for clarity.

To investigate how the structural differences induced by the aspartate-to-asparagine substitution in the variant enzyme could explain the mechanism of ceftazidime resistance and antibiotic “trapping,” molecular modeling of the Asp179Asn variant and KPC-2 in the presence of ceftazidime was performed (Fig. 6 and S6 to S8).

10.1128/mBio.00528-17.6 FIG S6  The Connolly surface representation viewed from the back of the Ω-loop of KPC-2 and Asp179Asn (D179N). Download FIG S6, TIF file, 0.5 MB. Copyright © 2017 Barnes et al. 2017 Barnes et al. This content is distributed under the terms of the Creative Commons Attribution 4.0 International license.

### (vii) Increased flexibility and mobility of the *Ω*-loop in the Asp179Asn variant.

Superimposition of the KPC-2 and Asp179Asn variant models (root mean square deviation [RMSD] of 0.6 Å) revealed that the salt bridge between Arg164 and Asp179 in KPC-2 is disrupted in the Asp179Asn variant (Fig. 1A and B). The Asp179 residue in KPC-2 forms a hydrogen bond network with Pro67, Leu68, Arg161, Asp163, and Arg164, but most of these interactions are absent in the variant (Fig. 1B). The disruption of the hydrogen bonds and salt bridge between Asp179 and Arg164 in the variant generates an “open channel” in the middle of the Ω-loop, enhancing the flexibility of this structure (Fig. 1A and B). Most of the side chains preserve their conformations (RMSD = ≤1 Å). However, the side chains of active-site residues are shifted by 0.5 to 1.5 Å and the active site in a Connolly representation is clearly deeper and wider, showing that the Asp179 variant has a more “open” conformation than KPC-2 (Fig. S6).

The analysis of trajectory generated during the 0.55-ns simulation of KPC-2 and Asp179Asn acyl-enzyme complexes with ceftazidime revealed increased mobility of individual residues of the Ω-loop for the Asp179Asn variant (average RMSD of 2 Å for KPC-2 compared to a 6 Å average for the variant) (Fig. 6A). The RMSD for the initial trajectory conformation of the KPC-2 Ω-loop increased from 1.5 Å for the first 120 ps to 3 Å (Fig. 6B). Impressively, the predicted movement of the Asp179Asn variant ranged from 1.4 Å for the first 40 ps to a 9 Å RMSD (Fig. 6B), showing in real time the increased mobility of the variant (Fig. 6C [KPC-2] vs. Fig. 6D [variant]).

### (viii) Structural impact on acylation.

Notably, a significant difference in hydrogen bond networks in Asp179Asn profoundly alters the position of the catalytic Ser70 in the oxyanion hole (Fig. S7). Ser70 in KPC-2 is oriented toward the oxyanion hole, forming hydrogen bonding interactions with Thr237 and a catalytic water; thus, the active site is “primed” and ready for catalysis. However, the hydroxyl group of Ser70 in the variant is shifted more than 1.5 Å and rotated more than 90° toward Asn170 and Glu166 (Fig. S7). The altered position of Ser70 supports alternative new hydrogen bond interactions with Lys73 and Asn170, which could make acylation more challenging for the variant.

10.1128/mBio.00528-17.7 FIG S7  KPC-2 (color by element) and Asp179Asn (D179N) variant (yellow) superimposition and hydrogen bond pattern. A and B green dotted lines denote hydrogen bonds. Yellow dotted lines indicate newly formed hydrogen bonds. The image in panel B is a magnified version of the image in panel A. Download FIG S7, TIF file, 0.3 MB. Copyright © 2017 Barnes et al. 2017 Barnes et al. This content is distributed under the terms of the Creative Commons Attribution 4.0 International license.

### (ix) Structural impact on deacylation.

The heat map of the hydrogen bonds generated during the molecular dynamics simulation (MDS) (Fig. S8A to F) suggests two possible pathways for ceftazidime deacylation by KPC-2. In the first pathway, Glu166 could act as a general base and activate the water molecule for proton transfer (for the first 10 ps, Glu166 forms a hydrogen bond with Ser70:Oγ) (Fig. S8A). Subsequently, the water is positioned between Glu166:Oε and Ser70:Oγ, allowing the hydrogen bond between Glu166 and the oxygen Oε and between Ser70 and the Oγ to alternate between the two hydrogen atoms of the water (Fig. S8B). Alternatively, K73 could serve as a proton donor (for *t* > 20 ps, the Lys73 is at a hydrogen bond distance from Ser70:Oγ) (Fig. S8C). The trajectory of the KPC-2 acyl-enzyme for the first 20 ps shows the catalytic water positioned at a hydrogen bond distance from Glu166 and Ser70:Oγ (Fig. 6E and S8B). Lys73 forms a hydrogen bond with Glu166 and is less than 3 Å distant from Ser70:Oγ.

10.1128/mBio.00528-17.8 FIG S8  Hydrogen bonds heat map for KPC-2 (A-C) and Asp179Asn (D179N) (D-F) generated during the 550-ps MDS. Download FIG S8, TIF file, 0.1 MB. Copyright © 2017 Barnes et al. 2017 Barnes et al. This content is distributed under the terms of the Creative Commons Attribution 4.0 International license.

In the Asp179Asn variant, the water molecule makes hydrogen bonds with Glu166:Oε but not with Ser70:Oγ (Fig. S8D and E). Instead, for the first 240 ps, Glu166 forms hydrogen bonds with Asn170 and water (Fig. 6E and S8D). Glu166 is 5 Å from S70:Oγ, and the water is positioned at 4.8 Å from Ser70:Oγ, unfavorable positioning for deacylation (Fig. S8E). Lys73 is oriented toward Asn132 and makes hydrogen bonds with Glu166 (Fig. S8F). After 240 ps, Glu166 and the catalytic water are favorably repositioned to make interactions with Ser70:Oγ to participate in deacylation (Fig. S8E).

Overall, these analyses suggest that Asp179Asn has structural perturbations in the active site and Ω-loop and associated hydrogen bond networks that result in decreased catalytic efficiency, which is consistent with the biochemical analysis that indicates that the Asp179Asn variant deacylates less rapidly and therefore acts as a “trap” for β-lactams.

### Conclusions.

Many single-amino-acid substitutions at positions 164 and 179 in KPC β-lactamase as well as other class A β-lactamases result in increased ceftazidime resistance and represent a clinical threat as a potential evolutionary adaptation to the widespread use of ceftazidime and other cephalosporins. One variant β-lactamase that is particularly notable is the KPC-2 Asp179Asn variant, as *E. coli* expressing *bla*_KPC-2 Asp179Asn_ demonstrated resistance not only to ceftazidime but also to other β-lactams and β-lactam–β-lactamase inhibitor combinations, including ceftazidime-avibactam. This laboratory analysis closely recapitulates the clinical observations being reported showing the emergence of KPC variants resistant to ceftazidime-avibactam (15). Strikingly, our data show that all strains containing variants at position 179 had elevated ceftazidime-avibactam MIC values. The clinical appearance of these variant β-lactamases poses a serious threat to ceftazidime-avibactam (2, 15). In this study, we also found that the drug MIC values of the KPC-2 variants in an isogenic background were decreased significantly with BAL30072 used as a monotherapy or with avibactam (as a model DBO) combined with imipenem, aztreonam, ceftaroline, or ceftriaxone. Although there was a significant reduction in MIC values when the carbapenems alone were tested, the Asp179Asn variant tested resistant to each of the carbapenems except meropenem (nonsusceptible).

Mass spectrometry showed a potential additional step in the enzymatic scheme for ceftazidime hydrolysis. Moreover, mass spectrometry data permit us to advance an explanation for why Asp179Asn shows enhanced ceftazidime resistance but not increased imipenem or aztreonam resistance. We propose that the 5-min “trap” of the acyl enzyme species serves as a “sink” for ceftazidime while still able being to hydrolyze it. However, this mechanism requires that the enzyme concentration be sufficient to “trap” all the ceftazidime, eliminating the intracellular pool of free ceftazidime available to bind to penicillin-binding proteins. In contrast, imipenem is “trapped,” but to a greater extent, and is stable at least three times longer, with the Asp179Asn-imipenem complex persisting as the most predominant species at 15 min. Aztreonam is also “trapped,” but less so, and the enzyme is subsequently inactivated by avibactam. Structural studies designed to identify the location and orientation of ceftazidime intermediates bound to Asp179Asn are needed to tease apart the details of this complex mechanism. However, it is very clear that the chemical nature of the ceftazidime adducts bound to Asp179Asn changes with time and that secondary-reaction chemistry is under way during catalysis.

Consistent with the increase in ceftazidime resistance for the Asp179Asn substitution in KPC-2, enhanced ceftazidime resistance in the Asp179Asn variant has also been found with TEM-1 (21) and SHV-1 (22) β-lactamases, indicating that the increased ceftazidime resistance caused by perturbations at the 179 position is a relatively global phenomenon for class A β-lactamases. However, the resulting effects seen in the kinetic characteristics of the Asp179 variants are substrate and enzyme specific. For example, the increased catalytic efficiency of the Asp179Asn variant in TEM was selective for ceftazidime among the nine substrates tested and correlated to increased affinity (21). We found that, relative to KPC-2 β-lactamases, the Asp179Asn variant demonstrated decreased hydrolysis of ceftazidime and imipenem and enhanced affinity for all substrates tested (aztreonam, ceftazidime, ceftaroline, ceftriaxone, ceftolozane, imipenem, and BAL30072).

Reduced activity in the Asp179Asn variant of KPC-2 parallels that of the Asp179Asn variant called P54 in PC-1 in *Staphylococcus aureus* due to disorder of the Ω-loop induced by dissociation of the salt bridge with Arg164 (23). That study found that the variant β-lactamase has an alternative interaction between Asp179Asn and Ala69 not found in PC-1. In addition, Stojanoski et al. showed that mutations in the TEM-1 Ω-loop induced conformational changes that permitted the subsequent enlargement of the active site to accommodate the large size of ceftazidime (increased burst kinetics) (24). Likewise, we found a correlation between a larger size in the R1 side group of the cephalosporins and increased drug MIC values for the Asp179Asn variant. These data are instrumental in designing effective inhibitors and pairing them with the most efficacious partner.

We found that BAL30072 shows promising activity against *E. coli* containing KPC-2 and all variants at position 179. These data raise the possibility that antibiotics such as monosulfactams may be optimally suited to pairing with DBO inhibitors for resistant strains. Indeed, several studies have already assessed the use of BAL30072 in combination therapy (25–28).

In closing, the analyses of Asp179 variants of KPC-2 showed that novel and catalytically versatile β-lactamases are emerging in the clinic and present an unprecedented challenge to drug development. The results from our biochemical and molecular studies reveal the basis of this unwelcome phenotype and point to rational approaches to overcome this resistance.

## MATERIALS AND METHODS

Ampicillin (catalog no. A9518), piperacillin (catalog no. P8396), ceftriaxone (catalog no. C5793), cephalothin (catalog no. C4520), potassium clavulanate (catalog no. 33454), cefotaxime (catalog no. C7912), and chloramphenicol (catalog no. R4405) were purchased from Sigma-Aldrich. Ceftazidime was procured from Sigma (catalog no. C3809) and Research Products International (catalog no. 33527), and the products from the two sources were used interchangeably throughout the experimentation. Imipenem was obtained from USP (catalog no. 1337809) and from the commercial source (pharmacy). Sulbactam was bought from Astatech. Tazobactam (catalog no. 15141) and aztreonam (catalog no. 15151) were purchased from Chem-Impex International. Ceftolozane-tazobactam, cefepime, meropenem, ertapenem, and doripenem were obtained from their commercial sources. Ceftaroline was provided by Allergan. Nitrocefin (catalog no. BR0063G) was purchased from Oxoid. Avibactam was purchased from Advanced ChemBlocks (catalog no. R16073).

### Site-saturation mutagenesis.

*Escherichia coli* containing *bla*_KPC-2_ in pBR322-*catI* vector was a gift from Fred Tenover (previously of the Centers for Disease Control and Prevention, Atlanta, GA) (29). For 16 of the 19 amino acid substitutions, mutagenesis was performed at nucleotides corresponding to position 179 in *bla*_KPC-2_ in the pBR322-*catI* plasmid using degenerate primers and a QuikChange site-directed mutagenesis kit (Agilent Technologies; catalog no. 200518-5) per the manufacturer’s instructions. Resulting plasmids were transformed into *E. coli* DH10B Electromax cells (Invitrogen).

The *bla*_KPC-2 Asp179Glu_, *bla*_KPC-2 Asp179Iso_, and *bla*_KPC-2 Asp179Ser_ genes with a ribosomal binding site (nucleotides 236 to 279) from pET24a+ vector positioned upstream of the *bla* gene and flanked by XbaI and BamHI restriction sites in the pBluescript II SK vector were purchased from Celtek Genes (Franklin, TN). The *bla*_KPC-2_ gene was amplified by PCR (Promega master mix) using T7 and M13 primers and was subsequently cloned into pCR-XL vector using a Topo XL PCR cloning kit (Invitrogen catalog no. 1647751). After electroporation into *E. coli* DH10B Electromax cells, plasmid from these cells was digested with XbaI and BamHI and ligated into pBC SK(+) vector. All nucleotide sequences corresponding to amino acid substitutions at position 179 in the *bla*_KPC-2_ gene were confirmed by sequencing (Molecular Cloning Laboratories, McLab, South San Francisco, CA) using *bla*_KPC-2_ primers.

### Expression and purification of KPC-2 and Asp179Asn.

The KPC-2 and Asp179Asn variant β-lactamases were purified from *E. coli* Origami 2 DE3 (Novagen) cells carrying the pET24a(+)*bla*_KPC-2_ or pET24a(+)*bla*_KPC-2 Asp179Asn_ plasmid as previously described for KPC-2 (7). Single colonies were used to initiate overnight growth of 5-ml cultures, and ~1.5 ml was used to start a 50-ml overnight culture. An overnight culture (10 to 12 ml) was added to each flask of 500 ml of super optimal broth (SOB), grown at 37°C to an optical density at λ_600_ (optical density at 600 nm [OD_600_]) of approximately 0.6 to 0.8, and induced with 0.5 mM isopropyl β-d-1-thiogalactopyranoside (IPTG) for a minimum of 3 h to express the β-lactamase. The cells were pelleted and frozen at − 20°C for ≥12 h prior to lysis in 50 mM Tris HCl buffer (pH 7.4) containing 40 mg/ml lysozyme, 0.1 mM magnesium sulfate, 250 U Benzonase nuclease, and 1 mM EDTA. The supernatant was further purified by preparative isoelectric focusing, eluted from a Sephadex column with 50 mM Tris-HCl (pH 8.8), subjected to sterile filtration, and purified once again using fast protein liquid chromatography (FPLC) and a HiTrap Q anion exchange chromatography column (GE Healthcare Life Sciences catalog no. 17-1154-01). The final sample of protein was concentrated using centrifugal filter units with a molecular weight cutoff of 10,000 (Millipore). A final 25% concentration of glycerol was added to the protein before the reaction mixture was frozen and stored at −20°C. The purity of the proteins was assessed by quadrupole time of flight (Q-TOF) mass spectrometry (see below). Protein concentrations were determined by measuring absorbance at a wavelength of λ_280_ and using the protein’s extinction coefficient (Δε; 39,545 M^−1^ cm^−1^) obtained using the ProtParam tool at the ExPASy Bioinformatics Resource Portal. All experiments using purified Asp179Asn were performed with multiple batch purifications.

### Whole-cell viability assays.

Mueller-Hinton (MH) agar-dilution MIC measurements were performed according to Clinical and Laboratory Standards Institute (CLSI) guidelines as previously described (12, 30). The MICs are reported as the concentrations at which bacterial growth was no longer observed. Avibactam was tested at a constant 4 µg/ml in combination with its respective antibiotic partners. All MIC measurements were performed at least three times.

### Protein expression.

Immunoblotting was performed to assess protein expression in whole-cell preparations (9) and periplasmic extracts (12) as previously described. To prepare whole-cell lysates, cells were grown to an OD_600_ of 0.7 to 0.8 using chloramphenicol to maintain the plasmid. One OD_600_ unit of cells was pelleted at 10,000 rpm for 5 min. The supernatant was removed, and the pellet was frozen at −20°C. Each pellet was resuspended directly in 50 μl of 5× sodium dodecyl sulfate-polyacrylamide gel electrophoresis (SDS-PAGE) loading dye and boiled for 10 min. Samples were subjected to vigorous vortex mixing. A volume of 10 μl of cell suspension was loaded onto each lane of a 10% SDS gel.

For periplasmic extracts, bacterial cultures were first cultured and stamped on MH agar plates per the CLSI guidelines for MIC determinations. Each culture was scraped from the MIC plate containing 2 µg/ml ceftazidime, resuspended in 1 ml of phosphate-buffered saline (PBS), and pelleted at 10,000 rpm for 5 min. Each cell pellet was lysed in 100 μl 50 mM Tris-HCl (pH 7.4) mixed with lysozyme, benzonuclease, MgSO_4_, and EDTA on a shaking platform. The protein content of cleared supernatant was determined using the Bio-Rad protein assay. Twenty micrograms of each extract was mixed with 5× SDS-PAGE loading dye, subjected to vortex mixing, and boiled for 10 min. A volume of periplasmic extract was loaded onto each lane of a 10% SDS-PAGE gel.

The samples on the SDS-PAGE gel were transferred to a polyvinylidene fluoride (PVDF) membrane, and nonspecific binding sites on the membrane were blocked for at least 1 h with 5% milk–25 mM Tris-buffered saline (TBS) (pH 7.4). The membrane was probed using an anti-KPC-2 antibody (1:5,000) (9) and anti-DnaK (*E. coli* monoclonal antibody [MAb]; Enzo Life Science catalog no. ADI-SPA-880) (1:30,000) as a protein loading control with 5% milk–TBS for 3 h at room temperature (or overnight at 4°C). After at least 10 washes in TBS–0.05% Tween 20 (TBS-T) for 10 min per wash, secondary antibodies (protein goat horseradish peroxidase [HRP; Bio-Rad catalog no. 170-6425] and goat anti-mouse IgG-HRP [Santa Cruz catalog no. sc-2005]) were used at 1:10,000 and 1:30,000, respectively, with 5% milk–TBS for 1 h at room temperature. The Western blot was washed again in TBS-T at least 10 times for 10 min each time and developed with an Amersham Prime ECL reagent kit (GE Healthcare catalog no. RPN2232) and a Fotodyne Luminary/FX workstation imaging system.

### Mass spectrometry.

Five micrograms of β-lactamase was incubated with substrate (ceftazidime, ceftaroline, ceftriaxone, imipenem, or aztreonam) and/or inhibitor (avibactam or BAL30072) at a molar ratio of 1:1 in sterile 10 mM phosphate-buffered saline (PBS) at pH 7.4 for a total reaction volume of 20 μl for the times indicated in the figures. Reactions were quenched with 10 μl acetonitrile and added to 1 ml 0.1% formic acid–water. Samples were analyzed using Q-TOF Waters Synapt-G2-Si and a Waters Acquity ultrapressure liquid chromatography (UPLC) BEH C_18_ column (1.7-µm pore size; 2.1 by 50 mm). MassLynx V4.1 was used to deconvolute protein peaks. The tune settings for each data run were as follows: capillary voltage at 3.5 kV, sampling cone at 35, source offset at 35, source temperature of 100°C, desolvation temperature of 500°C, cone gas at 100 liters/h, desolvation gas at 800 liters/h, and nebulizer bar at 6.0. Mobile phase A was 0.1% formic acid–water. Mobile phase B was 0.1% formic acid–acetonitrile. The mass accuracy for this system is ±5 Da.

### Steady-state kinetics.

Steady-state kinetic parameters were determined by using an Agilent 8453 diode array spectrophotometer at room temperature. Each assay was performed in 10 mM PBS at pH 7.4. Ceftazidime (25 µM) was incubated with 1 µM KPC-2 or Asp179Asn, and hydrolysis was measured for 60 minutes. Similarly, imipenem (100 µM) hydrolysis was performed with 0.5 µM KPC-2 or Asp179Asn β-lactamases. Competitive inhibition of NCF (50 µM) hydrolysis was also performed using 7 nM KPC-2 or 425 nM Asp179Asn and various concentrations of ceftazidime.

### Pre-steady-state stopped-flow kinetics.

For ceftazidime hydrolysis, 2.0 μM β-lactamase was incubated with 25 μM ceftazidime–10 mM sterile PBS (pH 7.4) at 25°C on an Applied Photophysics SX20 stopped-flow spectrophotometer (260-nm wavelength) using ProData SX software.

### Molecular modeling and docking.

A structural representation of the Asp179Asn variant of KPC-2 β-lactamase was generated using the crystal coordinates of KPC-2 (PDB: 2OV5) and Discovery Studio 4.1 (DS 4.1; Acclerys Inc., San Diego, CA) molecular modeling software as previously described (7, 8). The crystallographic water molecules were maintained during modeling. The KPC-2 β-lactamase structure and the variant model were solvated and minimized to an RMS value of 0.03 Å using a conjugate gradient method. To assess the stability of the models and possible conformational changes, molecular dynamics simulation (MDS) was conducted on the apo-enzymes for 0.55 ns.

Ceftazidime and acyl-ceftazidime were constructed using the Fragment Builder tools and minimized using a Standard Dynamics Cascade protocol of DS. The intact ceftazidime and acylated ceftazidime were automatically docked into the active site of KPC-2 and the Asp179Asn variant using the CDOCKER module of DS. To obtain acyl-enzyme complexes, the most favorable pose of ceftazidime demonstrating anticipated active-site contacts (such as a short distance [2 to 3 Å] between Ser70:O and C7 of ceftazidime) was chosen. MDS was conducted for 550 ps on KPC-2 and Asp179Asn as apo-enzymes and acyl-ceftazidime complexes. The trajectories were saved every 2 ps and analyzed for hydrogen bond heat maps, distances, etc.
