# Supplementary figures and images for: Klebsiella pneumoniae Carbapenemase-2 (KPC-2), Substitutions at Ambler Position Asp179, and Resistance to Ceftazidime-Avibactam: Unique Antibiotic-Resistant Phenotypes Emerge from β-Lactamase Protein Engineering
Source: mBio. 2017 Oct 31;8(5):e00528-17. doi: 10.1128/mBio.00528-17 (PMC5666153; doi:10.1128/mBio.00528-17)

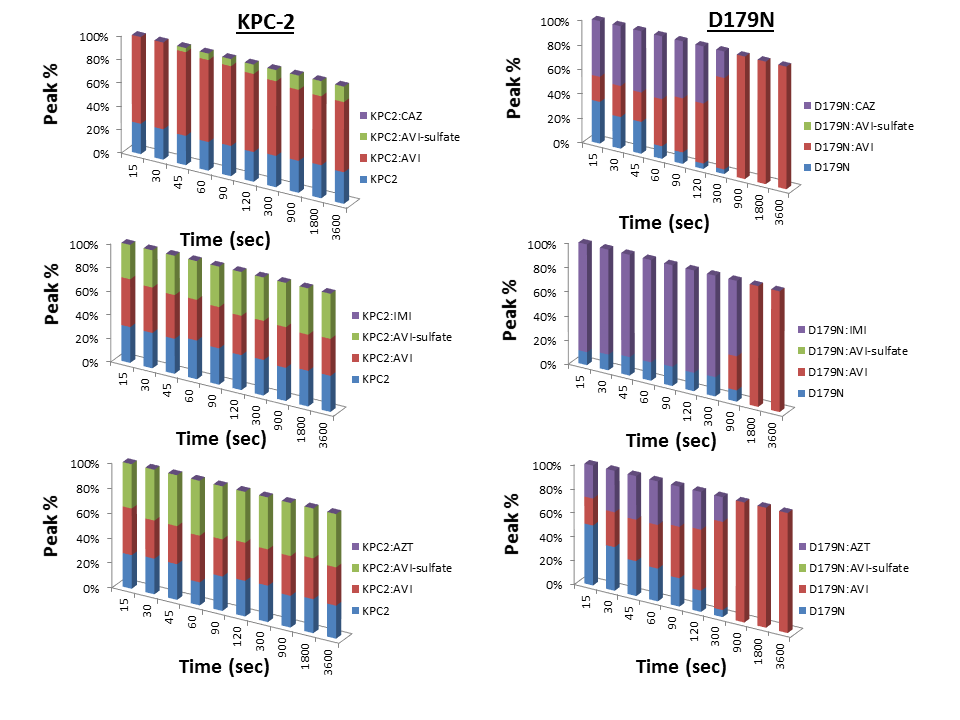

Supplement: FIG S1 [file mbo005173548sf1.tif]

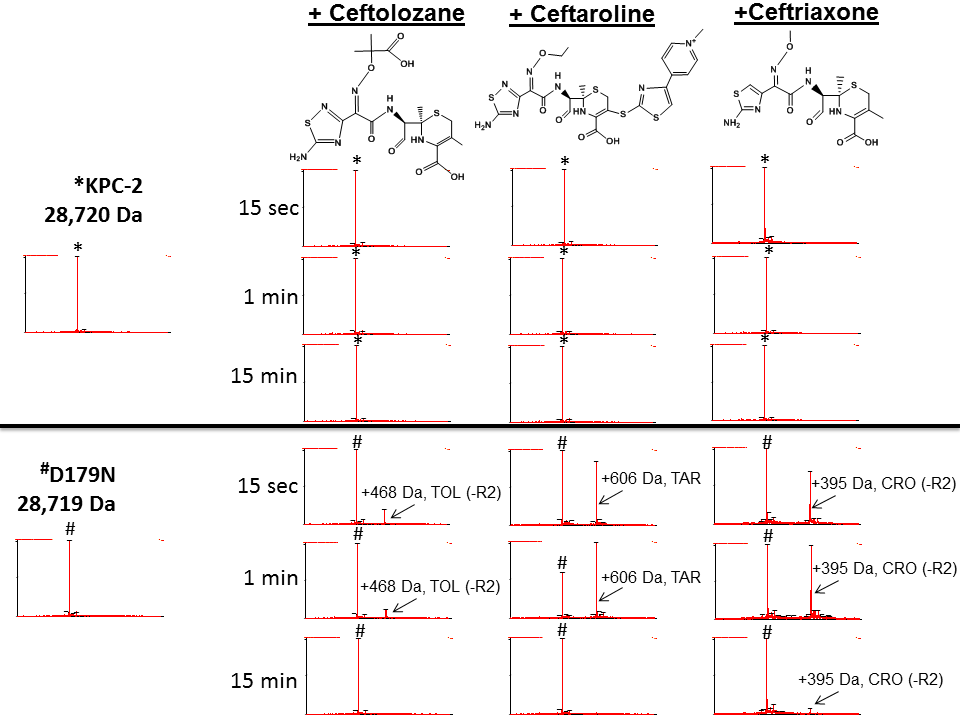

Supplement: FIG S2 [file mbo005173548sf2.tif]

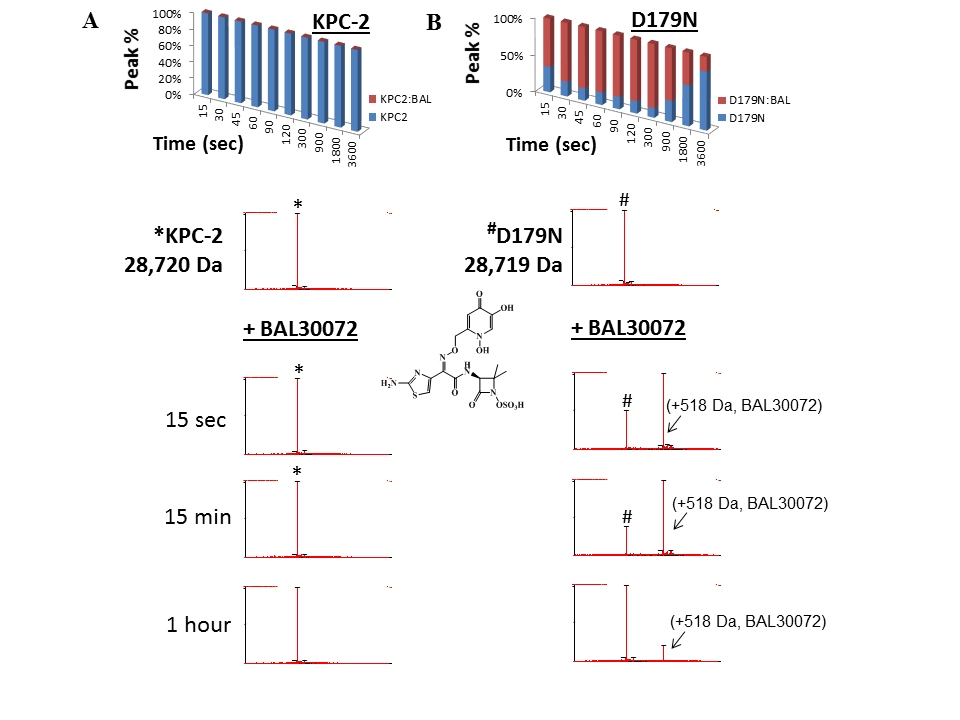

Supplement: FIG S3 [file mbo005173548sf3.tif]

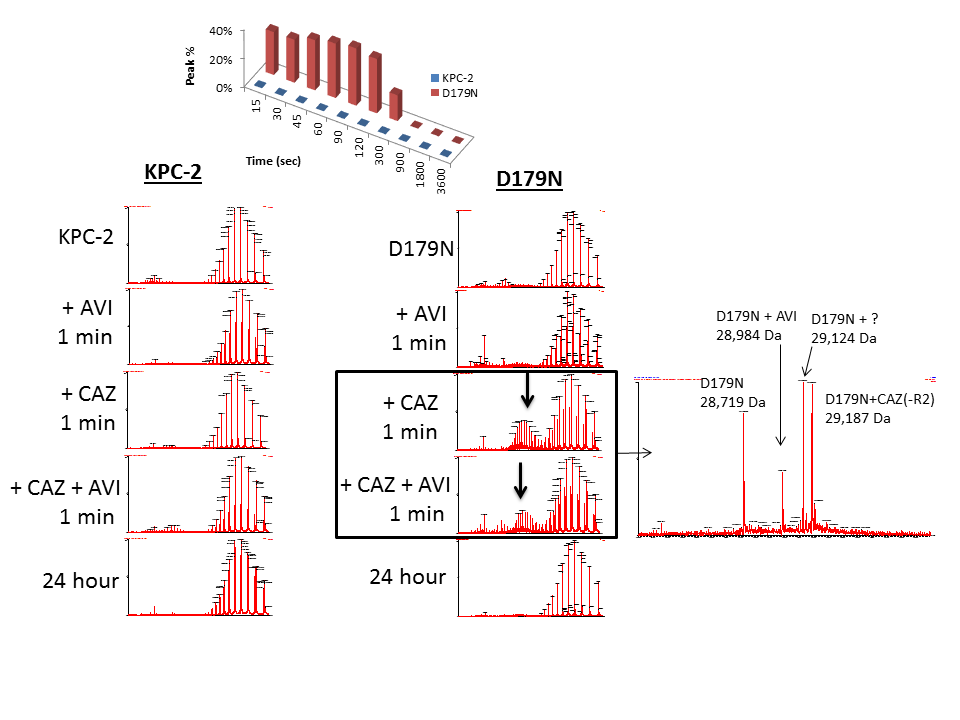

Supplement: FIG S4 [file mbo005173548sf4.tif]

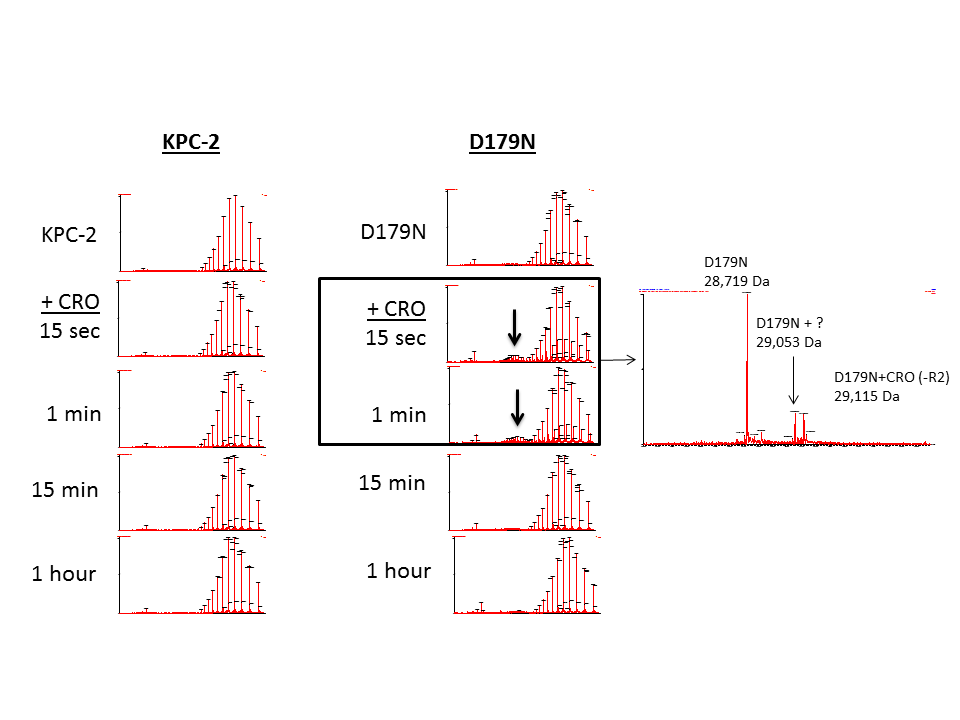

Supplement: FIG S5 [file mbo005173548sf5.tif]

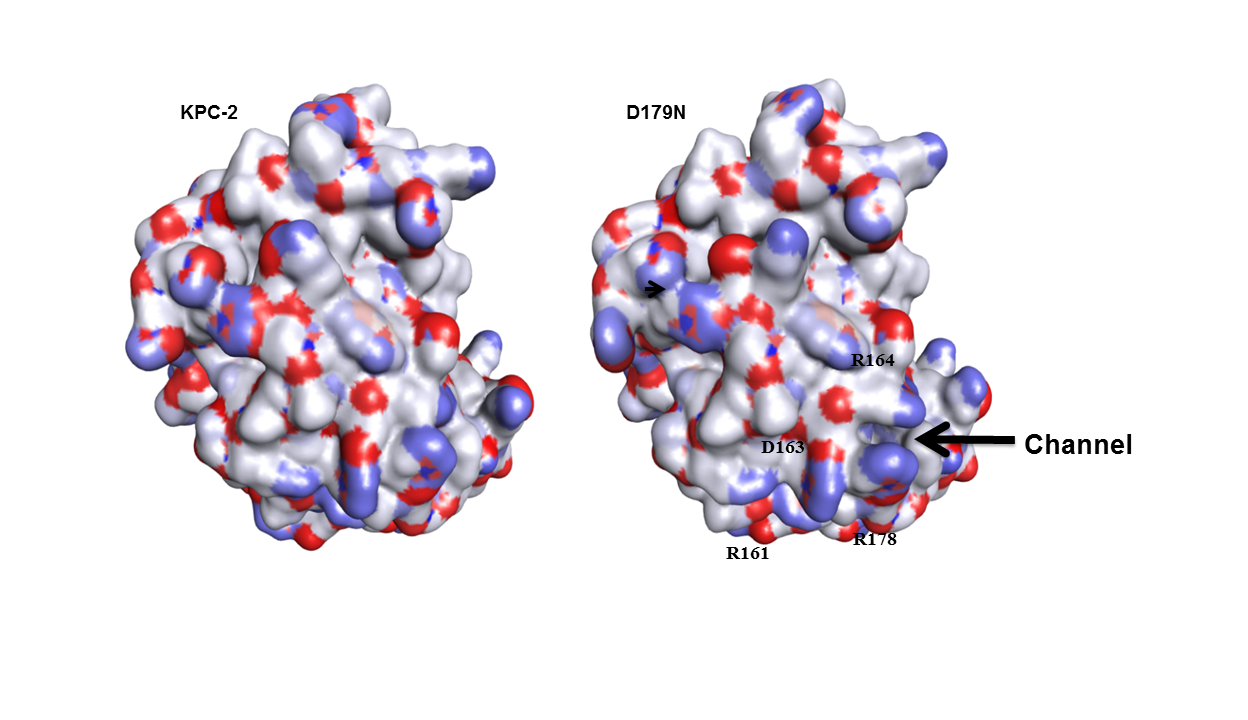

Supplement: FIG S6 [file mbo005173548sf6.tif]

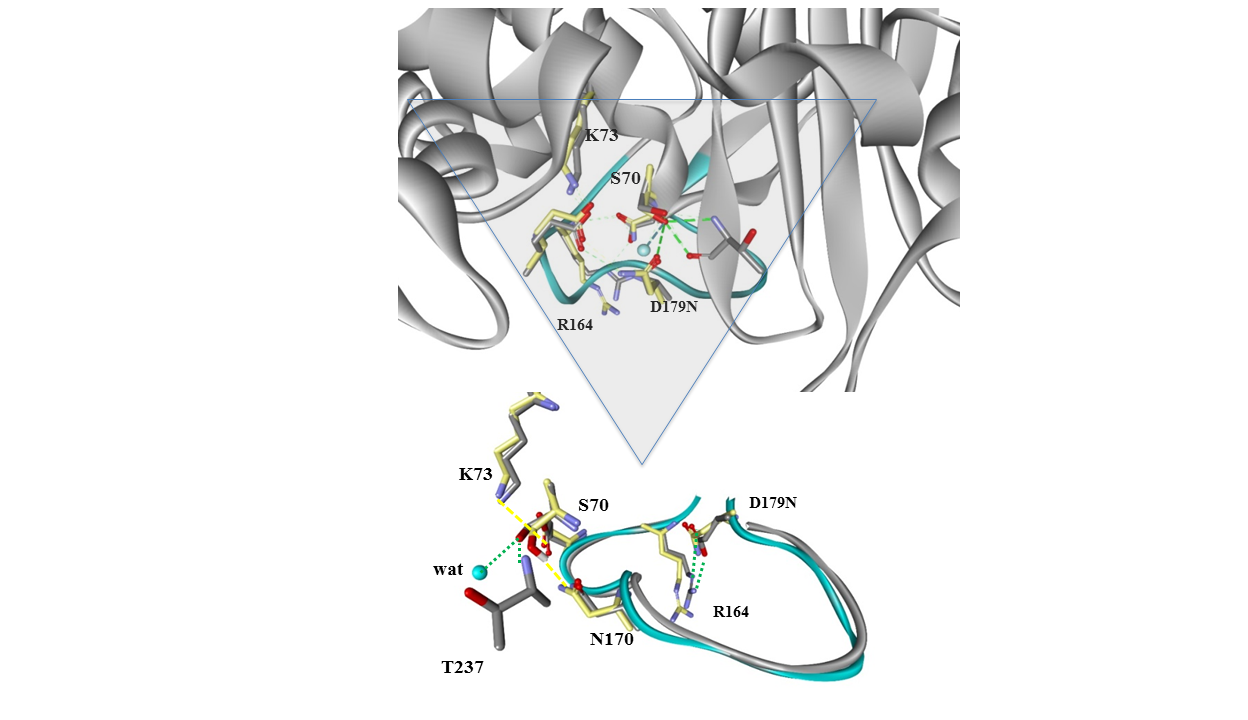

Supplement: FIG S7 [file mbo005173548sf7.tif]

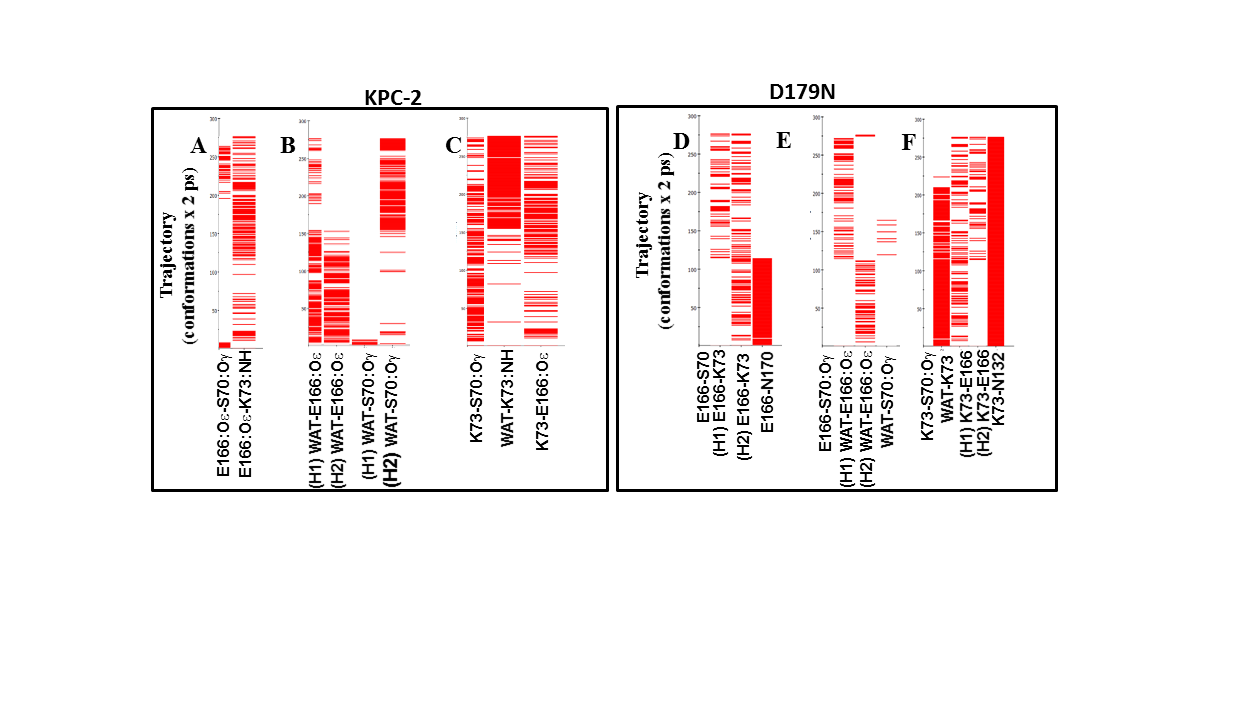

Supplement: FIG S8 [file mbo005173548sf8.tif]
